# Examining the Association of Rare Allelic Variants in Urate Transporters *SLC22A11*, *SLC22A13*, and *SLC17A1* with Hyperuricemia and Gout

**DOI:** 10.1155/2024/5930566

**Published:** 2024-01-06

**Authors:** Jiří Vávra, Kateřina Pavelcová, Jana Mašínová, Lenka Hasíková, Eliška Bubeníková, Aneta Urbanová, Andrea Mančíková, Blanka Stibůrková

**Affiliations:** ^1^Department of Cell Biology, Faculty of Science, Charles University, Prague, Czech Republic; ^2^Institute of Rheumatology, Prague, Czech Republic; ^3^Department of Rheumatology, First Faculty of Medicine, Charles University, Prague, Czech Republic; ^4^1st Department of Medicine, Department of Hematology; First Faculty of Medicine, Charles University and General University Hospital in Prague, Prague, Czech Republic; ^5^Department of Staphylococcal and Food-Borne Bacterial Infections, The National Institute of Public Health, Prague, Czech Republic; ^6^Department of Pediatrics and Inherited Metabolic Disorders, First Faculty of Medicine, Charles University and General University Hospital, Prague, Czech Republic

## Abstract

Genetic variations in urate transporters play a significant role in determining human urate levels and have been implicated in developing hyperuricemia or gout. Polymorphism in the key urate transporters, such as ABCG2, URAT1, or GLUT9 was well-documented in the literature. Therefore in this study, our objective was to determine the frequency and effect of rare nonsynonymous allelic variants of *SLC22A11*, *SLC22A13*, and *SLC17A1* on urate transport. In a cohort of 150 Czech patients with primary hyperuricemia and gout, we examined all coding regions and exon–intron boundaries of *SLC22A11*, *SLC22A13*, and *SLC17A1* using PCR amplification and Sanger sequencing. For comparison, we used a control group consisting of 115 normouricemic subjects. To examine the effects of the rare allelic nonsynonymous variants on the expression, intracellular processing, and urate transporter protein function, we performed a functional characterization using the HEK293A cell line, immunoblotting, fluorescent microscopy, and site directed mutagenesis for preparing variants *in vitro*. Variants p.V202M (rs201209258), p.R343L (rs75933978), and p.P519L (rs144573306) were identified in the *SLC22A11* gene (OAT4 transporter); variants p.R16H (rs72542450), and p.R102H (rs113229654) in the *SLC22A13* gene (OAT10 transporter); and the p.W75C variant in the *SLC17A1* gene (NPT1 transporter). All variants minimally affected protein levels and cytoplasmic/plasma membrane localization. The functional *in vitro* assay revealed that contrary to the native proteins, variants p.P519L in OAT4 (*p* ≤ 0.05), p.R16H in OAT10 (*p* ≤ 0.05), and p.W75C in the NPT1 transporter (*p* ≤ 0.01) significantly limited urate transport activity. Our findings contribute to a better understanding of (1) the risk of urate transporter-related hyperuricemia/gout and (2) uric acid handling in the kidneys.

## 1. Introduction

Uric acid is a catabolic product of purine metabolism in humans and higher primates (chimpanzees, orangutans, gibbons, and new world apes) who have lost urate oxidase activity [1, 2]. This water-soluble organic compound has a physiological role as an antioxidant, and it is thought that increased plasma urate levels (uricemia) caused an acceleration of brain development during evolution; furthermore, uric acid may help the immune system in recognizing injured or damaged cells [3–5]. In addition to its physiological roles, uric acid also has clinical significance. Increased plasma urate concentration (hyperuricemia) can cause cardiovascular disease [6], high blood pressure [7], and renal disease [8]. Hyperuricemia and gout have a significant impact on lipid metabolism, the most significant glycerophospholipid dysregulation was recently found in early onset patients ≤40 years [9]. Prevalence in the European adult population is estimated to 11.9%–25.0% for hyperuricemia and 0.3%–4.7% for gout, respectively [10]. Over the past decade, genome-wide association studies and meta-analyses have led to a massive increase in our knowledge of the common genetic variants that influence serum UA concentrations. However, knowledge of the extent to which genetic variants predict serum UA concentrations and the functional pathways through which they may influence serum UA levels remains limited.

**Figure 1:**
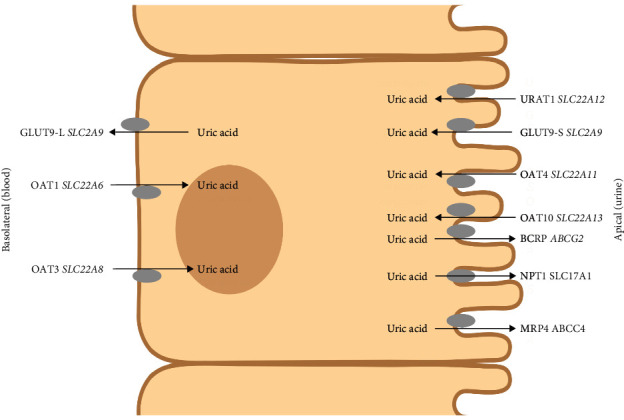
Scheme of renal tubular epithelial cells. Transporters OAT4 and OAT10 provide reuptake of urate from the luminal side of proximal tubules, while NPT1 is responsible for urate excretion across the luminal membrane of tubules into the urine. Created with BioRender.com.

Urate excretion or reabsorption in the proximal tubule of the nephron is managed by various transporters [11]—multimolecular complex “transportosome” that probably involves cooperation between multiple transporters, Figure 1. For urate excretion in the proximal tubule seems to be most important ABCG2 transporter (BCRP), which is localized on an apical membrane of human proximal tubule [12, 13]. It was shown that single nucleotide polymorphism (SNP) such as p.Q141K is associated with hyperuricemia and/or gout [14]. This mutation caused impaired transport function [15]. Similarly, p.Q126X allelic variant in this transporter abolished urate excretion [16]. Moreover, further SNPs were discovered in *ABCG2* which change the transport capacity for urate [17]. Other variants associated with hyperuricemia and gout were found in the genes encoding the transporters GLUT9 and URAT1 [18]. These SNPs are associated with hyperuricemia[19–21] and even more often with renal hypouricemia [22, 23]. URAT1 is urate transporter localized on the apical membrane of proximal tubular cells [24]. The association of p.C850G with gout has been described [25], but similarly as in the GLUT9, allelic variants seem to be more frequently associated with hypouricemia [26, 27]. In addition to these well-known and well-studied transporters in the renal epithelial cells, there are other proteins involved in urate handling in the kidney. In our last paper, we examinated rare variants in *SLC22A6* (OAT1) and *SLC22A8* (OAT3) and in this work we focus on another less examined urate transport proteins [28]. In Table 1, we present an overview of the influence of identified allelic variants in the urate trasportosome, which we published in our previous paper.

### 1.1. *SLC22A11* (OAT4)

Organic anion transporter 4 (OAT4) is a 550 amino acid protein encoded by the *SLC22A11* gene. The OAT4 protein is expressed in the apical membrane of the proximal tubules in the kidney as well as in the placenta [32, 33]. This protein functions as an organic anion/dicarboxylate exchanger [34] and as a low-affinity urate transporter [35]. Some studies have reported polymorphisms in *SLC22A11* associated with serum urate concentrations and gout [36–39]. Single amino acid substitutions, i.e., p.L29P and p.H469R, both of which decrease urate transport by the OAT4 transporter, were found in the healthy individuals [40].

### 1.2. *SLC22A13* (OAT10)

Organic anion transporter 10 (OAT10) is a 551 amino acid protein encoded by the *SLC22A13* gene. OAT10 was first discovered as the orphan transporter hORCTL3; its sequence was similar to the organic cation transporter family [41]. Protein OAT10 is highly expressed in the apical membrane of the proximal tubules in the kidneys, where it mediates urate reabsorption [42, 43]. This protein transports organic anions, such as urate, nicotinate, orotate, or para-aminohippurate. Urate is exchanged for OH-anions or organic anions such as lactate, pyrazinoate, or nicotinate [42, 44]. To date, there are no known variants of the *SLC22A13* gene associated with the risk of gout; on the other hand, the polymorphism rs117371763 (p.R377C) is associated with a reduction of gout risk [45].

### 1.3. *SLC17A1* (NPT1)

Sodium-dependent phosphate cotransporter type 1 (NPT1) is a 467 amino acid protein encoded by the *SLC17A1* gene. The NPT1 protein is expressed mainly in the kidneys and liver. NPT1 was initially discovered as a sodium phosphate cotransporter in the cortex of human kidneys [46]. NPT1 has also been shown to transport para-aminohyppurate, estradiol-17- *β*-glucuronide, and urate. The transporter is chloride sensitive and is located on the apical side of epithelial cells [47, 48]. The transport is dependent on the membrane polarization and is responsible for the urate excretion from cells [49]. The variant rs1165196 (p.I269T) in *SLC17A1* causes an increase in urate transport via NPT1 and, therefore, significantly reduces the risk of gout linked to renal underexcretion. This variant increases the urate transport capacity in uptake assays with *Xenopus* oocytes in a high-potassium buffer [48, 50]. In addition, the intronic variant rs1183201 in *SLC17A1* has been described as a variant significantly associated with gout [51].

This study examines six rare nonsynonymous variants in the *SLC22A11*, *SLC22A13*, and *SLC17A1* genes found in a cohort of 150 Czech patients of Caucasian origin with primary hyperuricemia and gout. This study finished the complex analysis concerning the identification of genetic variants, their influence, and mutual interactions of individual variants of urate transportosome on serum UA concentrations encompassing the detailed characterized cohort suffered from the primary hyperuricemia/gout.

## 2. Material and Methods

### 2.1. Subject

The primary cohort consisted of 36 patients with primary hyperuricemia (21 males/15 females) and 114 individuals with primary gout (100 males/14 females). We also analyzed a cohort of 34 pediatric patients with hyperuricemia and gout, 10 of whom were included in the primary cohort. We previously described this cohort in our publication focusing on pediatric patients [52]. Primary hyperuricemia was defined as serum uric acid levels (SUA) > 420 *μ*mol/L (7.0 mg/dL) in men and >360 *μ*mol/L (6.0 mg/dL) in women and children under 15 years. Primary hyperuricemia was confirmed with a second measurement at least 4 weeks after the first. Gouty arthritis was diagnosed according to the criteria of the American College of Rheumatology [53]. The control group consisted of 115 normouricemic subjects. All patient samples were collected from the Biobank of the Institute of Rheumatology in Prague. Patients with secondary gout and other disorders of purine metabolism manifesting as pathological SUA concentrations were excluded. All tests were conducted according to standards set by the institutional ethics committee. All procedures were performed in accordance with the Declaration of Helsinki.

### 2.2. Material

Plasmid DNA with cloned genes, turboGFP mouse monoclonal antibody (cat. TA150041, clone OTI2H8) was purchased from OriGene Technologies, Inc, USA. The following vectors were used: *SLC17A1* (cat. RG211006), *SLC22A11* (cat. RG206469), and *SLC22A13* (cat.RG222116) cloned in pCMV6-AC-GFP with C-terminal turbo GFP tag. The HEK293A cell line was purchased from ThermoFisher Scientific, USA (cat. R70507). DMEM, fetal bovine serum (FBS), and other cultivation media and supplements were also from the ThermoFisher Scientific. The secondary antibody conjugated with HRP (cat.A90-117P) was purchased from Bethyl-Fortis Life Science, USA. The *β*-actin primary antibody (clone 8H10D10) was purchased from Cell Signaling, USA. Cultivation plastic for cells was purchased from VWR. Radiolabeled uric acid (MC-1394) was purchased from the Hartman Analytic GmbH, Germany. Western blot material and all common chemicals were from Merck KGaA, Germany, or Penta s.r.o, Czech Republic. Primers for sequencing and mutagenesis were synthesized by Generi biotech, Czech Republic. For measurement of radioactivity, we used liquid scintillant Ultima Gold and a TriCarb 2900TR scintillation counter (USA).

### 2.3. PCR Amplification and Sequencing Analysis

Genomic DNA was isolated from EDTA-anticoagulated whole blood using GeneAll Exgene Blood SV mini kits (GeneAll, South Korea). All coding exons and exon/intron borders of the *SLC22A11*, *SLC22A13*, and *SLC17A1* genes were PCR amplified. The PCR products were confirmed using gel electrophoresis in 2% agarose gel and purified using PCR Cleanup Kits (Geneaid, Taiwan). Subsequent Sanger sequencing was performed using a BigDye Terminator v3.1 Cycle Sequencing kit (Applied Biosystems, USA) and CentriPure Dye Terminator purification 96-well plates (Gennaxon bioscience, Germany). DNA sequencing was carried out using an ABI PRISM 3100 Genetic Analyzer (Applied Biosystems, USA). The resulting sequences were evaluated using SeqMan Pro software and DNASTAR Lasergene (DNASTAR, Inc., USA).

### 2.4. Site-Directed Mutagenesis and Plasmid Preparation

First, we performed site-directed mutagenesis with a GENEART Site-directed mutagenesis system (Invitrogen). We used the standard protocol recommended in the kit manual. We used the primers listed in Table S1. The plasmids were multiplied in DH5*α* bacteria. For the preparation of pure plasmids, we used mini and maxi kits provided by Qiagen, Germany. Before transfection, each plasmid was sequenced to evaluate the correct sequences.

### 2.5. Cell Maintenance and Transfection

HEK293-A cells were maintained according to the usual protocol. The cells were cultivated in Dulbecco Minimal Eagle Medium (Gibco, 11965092), supplemented with 10% FBS (Gibco, 10270106), 1.0 mM pyruvate, 1.0% nonessential amino acids solution, and gentamycin 0.04 mg/mL. We passaged the cells with a 1% trypsin-EDTA solution when the cells grew to ca. 90% confluency. After thawing the aliquot, which had been stored in liquid nitrogen, we allowed the cells to grow for two passages and then used them in uptake assays.

### 2.6. Microscopy

We prepared cover glasses with 0.01% poly-L-lysine (Merck, Germany) in 12-well dishes. After this step, we seeded the cells with 0.05× 10^6^ cells per well. We transfected the cells at ca. 20% confluency, and after 24 hr, we fixed the cells with 4% paraformaldehyde for 10 min (after longer fixation, the tGFP signal was quenched, data not shown). We mounted the cover glasses with cells in Mowiol with DAPI 0.1 *µ*g/mL. The cells were observed using a Leica DM-6 fluorescence microscope.

### 2.7. Immunoblotting

We performed western blotting to detect the transfection efficiency of the different variants of each transporter with same method as in our previous paper [28]. We prepared a transiently transfected cell monolayer, as shown above. We collected the cells with a scraper into phosphate-buffered saline (PBS, pH = 7.4) and centrifuged them at 150x *g* for 5 min. After this step, we lysed the cells with a 10% SDS and 9% protease inhibitory cocktail (Merck, cat I3786). We resuspended cells in lysis solution immediately, and then we sonicated the samples with a UP50H (Hielscher) ultrasound homogenizer on ice (25 impulses per sample at 60% amplitude). Afterward, we mixed the samples with 2x Laemmli loading buffer (Merck, cat. S34701) and denatured them at 56°C for 60 min. The samples were loaded on an SDS-PAGE acrylamide gel (5% focusing and 10% separating gel). After separation and blotting on a PVDF membrane, we blocked the membrane with 5% nonfat milk for 1.5 hr and then incubated it with the primary antibody overnight at 4°C. We used the anti-tGFP antibody 1 : 1,000 and anti-CapZ antibody 1 : 1,000. Then, we incubated it with the secondary, HRP conjugated antibody 1 : 7,000 and detected the signal from the SuperSignal West Pico Plus Chemiluminescence substrate (Thermo Scientific, USA). The chemiluminogram was taken using a BioRad Chemidoc touch imaging system (Biorad, USA).

### 2.8. Uptake Assay

Cells from the storage bottle were passaged with 1% trypsin-EDTA solution, dissociated cells were added to fresh medium, and this suspension was centrifuged at 150x *g* for 5 min. After this step, cell pellets were diluted with fresh medium, and cells were plated in 12-well dishes at a concentration of 0.2 × 10^6^ cells per well. The cells were further cultivated for 48 hr and transfected with the polyethyleneimine (PEI) method after reaching about 75% confluency. For each 1.0 *µ*g of DNA, we used 2.6 *µ*g of PEI. The cells were further cultivated for 48 hr, followed by uptake assays. We used cell cultivation dishes covered with 0.01% poly-L-lysine (Merck, Germany). The uptake assay was performed according to our previous study and other published work [28, 54, 55]. First, we flushed out the cultivation medium and washed the monolayer twice with Hankʼs balanced salt solution (HBSS, NaCl 138.0 mM, KCl 5.0 mM; CaCl_2_ 1.0 mM, MgCl_2_ 0.5 mM, MgSO_4_ 0.4 mM; KH_2_PO_4_ 0.4 mM; NaHCO_3_ 4.0 mM; Na_2_HPO_4_ 0.3 mM; glucose 5.6 mM, pH = 7.4). After this step, cells were preincubated with HBSS for 15 min at 37°C. Subsequently, the HBSS was removed, and 30 *µ*M 14C radiolabeled uric acid dissolved in HBSS was added to three wells from each variant and cultivated at 37°C. We used different cultivation times for the isotopes—5 min for SLC22A13, 20 min for SLC22A11, and 30 min for SLC17A1. The fourth well from each variant was used for measuring protein concentrations and was treated the same way but not with radiolabeled uric acid. After incubation, the radioactive HBSS was removed, and the washed cell monolayer was kept on ice and rinsed three times with ice-cold HBSS. After washing the cells, they were lysed in 0.15 M NaOH for 2 hr with mild shaking; the cultivation dishes were kept on ice. The cells transfected with *SLC17A1* were treated the same, but we used depolarizing HBSS (HBSS 142 mM potassium gluconate; 5.0 mM sodium gluconate; 1.8 mM CaCl_2_; 1.0 mM MgCl_2_; 5.0 mM HEPES; pH = 7.4) instead of HBSS. After completion of lysis, the lysate was collected into scintillation vials, neutralized with HCl, and Ultima Gold liquid scintillant was added. After measuring isotope activity with a TriCarb 2900 TR (1 min of premeasuring and 5 min of measuring), protein samples were stored at −80°C. For further measurement of these protein samples, we used Bradford assay with a Biorad Start Bradford dye reagent (Biorad, USA).

### 2.9. Evolutionary Alignment

We used an Uniprot alignment tool. In this application, we used protein sequences obtained from an Uniprot database [56]. In Figure S1, we marked evolutionary fully conserved amino acid residues with gray color and similar amino acid residues with black rectangles without fill.

### 2.10. Statistical Analysis

The scintillation counter provided data in counts per minute, which we converted to disintegrations per minute (DPM) with a quenching correction. Uric acid uptake was in pmol per mg of protein per minute. Finally, we expressed the uptake as a percentage of the wild-type protein variant since this form is better for understanding, and the results were not affected by the current condition of the cells. We repeated the uptake assay and protein concentration measurements three times independently; the cells were from different frozen aliquots. In each repetition, we have three wells for uptake measurement (each with 2.0 × 10^6^ cells) for each group, i.e., control, wild type, and allelic variants. Then, we obtained three values of CPM for each of this variant. From all these measurements, we calculated the mean and the standard deviation. We used the Studentʼs *t*-test for two samples with different variances for statistical analysis. We plotted the data into graphs, where the statistical significance expressed as *p*-values are marked by asterisks ( ^*∗*^ for *p* ≤ 0.05 and  ^*∗∗*^ for *p* ≤ 0.01). The statistical analysis and graph plotting was performed in Microsoft Excel 2019 MSO (Microsoft, USA).

## 3. Results

### 3.1. Subjects

The cohort used for this study was identical to our previous study. The main demographic and biochemical parameters of the patients are summarized in Table 1 in our previous paper [28]. The cohort consisted of 150 patients—114 with gout (100 men and 14 women) and 36 with primary hyperuricemia (21 men and 15 women). The familial occurrence was 30.7% in gout and 19.4% in hyperuricemia. The median age/age of onset was 59/45 years for gout patients and 55.5/48 years for hyperuricemia patients. The median of serum uric acid values and fractional excretion of uric acid at the time of examination was 371 *µ*mol/L and 3.4 for gout patients and 411 *µ*mol/L, and 3.5 for hyperuricemia patients.

### 3.2. Sequencing Analysis

In the *SLC22A11* gene (Table 2), we found missense variants p.V202M (rs201209258), p.R343L (rs75933978), and p.P519L (rs144573306). Furthermore, in this gene, we detected two rare synonymous variants p.T110T (rs774860411) and p.L496L (rs753269187). In analyzed intron regions, we discovered c.497 + 85A > G (rs2277312), c.1058 + 53A > C (rs71456318), and c.1589 + 54T > A (rs185640375). In the *SLC22A13* gene (Table 2), we also identified two missense variants, p.R16H (rs72542450) and p.R102H (rs113229654), and further two rare synonymous variants, p.A53A (rs9842091) and p.P186P (rs146083340). Besides these variants, we described several variants in analyzed intron regions c.1022 + 31C > G (rs41285121), c.1346 + 86A > G, c.1346 + 107G > A (rs551131182), c.1346 + 139C > T (rs2236631), c.1346 + 164G > A (rs1456539831), and c.1346 + 208C > T (rs181912533). In the *SLC17A1* gene (Table 2), we discovered one rare nonsynonymous variant, p.W75C (rs149708935), with minor allele frequency (MAF) <0.1% in Europeans, and one common nonsynonymous variant, p.T269I (rs1165196), with MAF 56.5% in Europeans. In addition, we detected seven variants in analyzed intron regions c.207 + 115C > T (rs115398536), c.207 + 40C > T (rs373732735), c.208− 14C > T (rs200114666), c.898− 71T > C (rs10498730), c.1269 + 61A > G (rs1165210), c.1179 − 111C > T (rs1165209), and c. ^*∗*^ 2 + 79C > T (rs1165215). We used some *in silico* model (SIFT, PolyPhen, CADD, REVEL, MetaLR, and MutationAssesor) for predicting impact of examined allelic variants [57]. We obtained deleterious or damaging in case of p.P519L (OAT4), p.W75C (NPT1), and p.V202M (OAT4). Surprisingly for p.R16H, we did not obtain any damaging prediction (Table S2).

### 3.3. Functional Study

#### 3.3.1. *SLC22A11* (OAT4)

**Figure 2:**
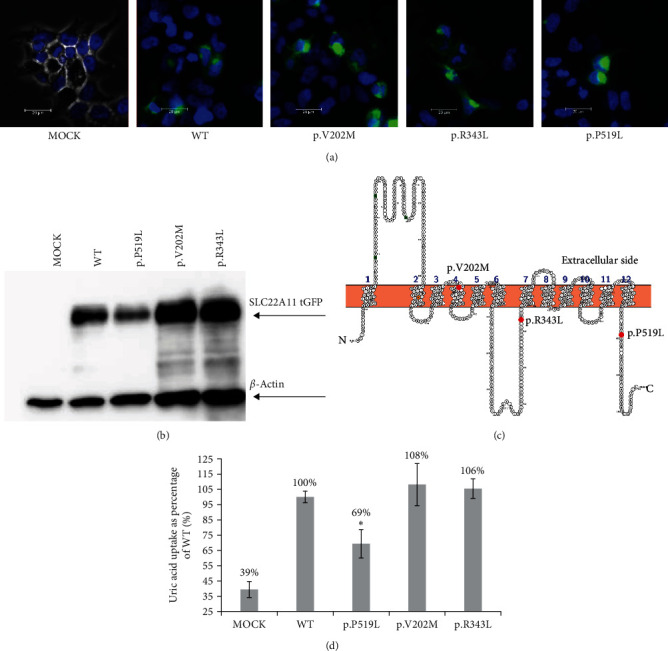
(a) Expression of OAT4 (*SLC22A11*) and its allelic variants in the HEK293A cell line. Wild-type protein was tagged with a C-terminal tGFP tag, and cells were transiently transfected by polyethyleneimine lipofection. Samples were fixed with 4% paraformaldehyde, and pictures were taken with a Leica DM6 microscope at 400x magnification. Plasma membrane localization was observed in all variants. Cell nuclei were stained with DAPI (blue). (b) Western blot of OAT4 allelic variants. Expression of the protein was detected with tGFP antibody and anti-*β*-actin antibody as a loading control. (c) Predicted structure of OAT4 protein visualized in Protter [58]. Allelic variants are marked with red dots. (d) Uptake study with 30 *µ*M 14C uric acid with 20-min incubation. The intracellular uptake of urate is expressed as a % of the wild-type uptake, *n* = 3,  ^*∗*^means *p* ≤ 0.05 (Students *t*-test).

We identified three allelic variants of the OAT4 transporter—p.V202M, p.R343L, and p.P519L (Table 2). We performed an uptake assay with 30 *µ*M 14C labeled uric acid with 20 min of incubation at 37°C in HBSS buffer (Figure 2(d)). As a baseline, we used uric acid uptake by the OAT4 wild-type protein (100%). The uptake of urate by the negative control (MOCK, cells transfected only with water instead of the plasmid with the transporter gene) was 39.4% of the wild type. We think this uptake was caused by endogenously expressed transporters and a channel in the HEK293A line. Substitution with p.P519L in our uptake assay decreased urate uptake to 69.4% of the wild-type uptake. This decrease in uptake was significant, with a *p*-value <5%. Substitution with p.V202M produced a nonsignificant increase to 108.1% of the wild type. Because the standard deviation of the value of this transporter is relatively high, we suspect that the transport activity of the transporter with this variant is very similar to the wild type. Finally, p.R343L nonsignificantly increased the uptake to 105.5% of the wild type. As with the p.V202M variant, the standard deviation of this value implies that the uptake is probably similar to the wild type. Further, we analyzed the membrane localization using microscopy (Figure 2(a)), and based on this we suspect that all the variants of this transporter have a cell membrane localization. We also analyzed protein expression using western blot (Figure 2(b)). In MOCK, we did not obtain any band for the tGFP tag. In the wild type and p.P519L, we obtained a band with a similar density to p.V202M and p.R343L, which had a stronger band than the wild type and p.P519L. Both variants (p.V202M and p.R343L) had a concurrently denser band of loading control (*β*-actin) than the other two variants (wild type and p.P519L). This result suggests that all transporter variants are expressed at similar levels. The p.R343 is evolutionarily conserved among the representative models in higher primates (*Homo sapiens* and *Pan troglodytes*), ruminants (*Bos taurus*), artiodactyla (*Sus scrofa*), and birds (*Gallus gallus*). Similarly, the p.F519 is conserved in all mammalian models, and birds but not in the ruminantia, where it is substituted with leucine. The p.V202 is conserved only in higher primate models, but in ruminantia, artiodactyla, and bird models, it is substituted with leucine (Figure S1).

#### 3.3.2. *SLC22A13* (OAT10)

**Figure 3:**
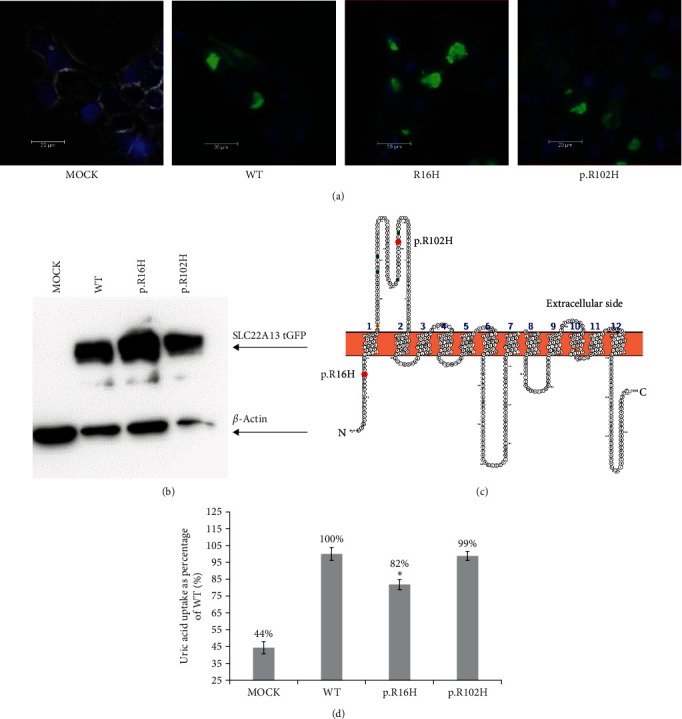
(a) Expression of OAT10 (*SLC22A13*) and its allelic variants in HEK293A cells line. Wild-type protein was tagged with a C-terminal tGFP tag, and cells were transiently transfected by polyethyleneimine lipofection. Samples were fixed with 4% paraformaldehyde, and pictures were taken with a Leica DM6 microscope at 400x magnification. Plasma membrane localization was observed in all variants. Cell nuclei were stained with DAPI (blue). (b) Western blot of OAT10 allelic variants. Expression of the protein was detected with tGFP antibody and anti-*β*-actin antibody as a loading control. (c) Predicted structure of OAT4 protein visualized in Protter [58]. Allelic variants are marked with red dots. (d) Uptake study with 30 *µ*M 14C uric acid with 5-min incubation. The intracellular uptake of urate is expressed as a % of the wild-type uptake, *n* = 3,  ^*∗*^means *p* ≤ 0.05 (Student's *t*-test).

In OAT10, we identified two allelic variants—p.R16H and p.R102H (Table 2). We performed the uptake assay using the same conditions as the OAT4 transporter and its variants but with different incubation times—5 min with the uric acid isotope (Figure 3(d)). Similarly, we chose wild-type uptake of uric acid as the baseline. The uptake of urate by the negative control (MOCK) was 44.3% of the uptake by the OAT10 wild type. The uptake by p.R16H significantly decreased to 81.8% of the wild type with a *p*-value <5%. Finally, p.R102H nonsignificantly decreased the uptake to 98.8% of the wild type, but based on the standard deviation, we suspect that transport activity was not affected. We performed fluorescent microscopy of protein localization on the cell membrane and concluded that all variants are localized on the cell membrane (Figure 3(a)). Similarly, as in the previous transporter, we examined the protein expression of the transporter using western blot (Figure 3(b)). In MOCK, we did not detect any tGFP signal (only loading control *β*-actin). We observed a similar band density in the wild type and p.R16H. In p.R102H, we observed a lower density, which corresponded to the density of the loading control (*β*-actin). We concluded that all variants were expressed in cells at similar levels. Subsequently, we performed an evolutionary analysis of the protein sequence of the transporter (Figure S1). Arginine 16 is conserved in all mammalian model species but not in the model representative of birds—chicken (*Gallus gallus*); in this species we found p.R16P and p.R102G substitutions. Surprisingly, we identify R102H substitution in rhesus macaques (*Macaca mulatta*).

#### 3.3.3. *SLC17A1* (NPT1)

**Figure 4:**
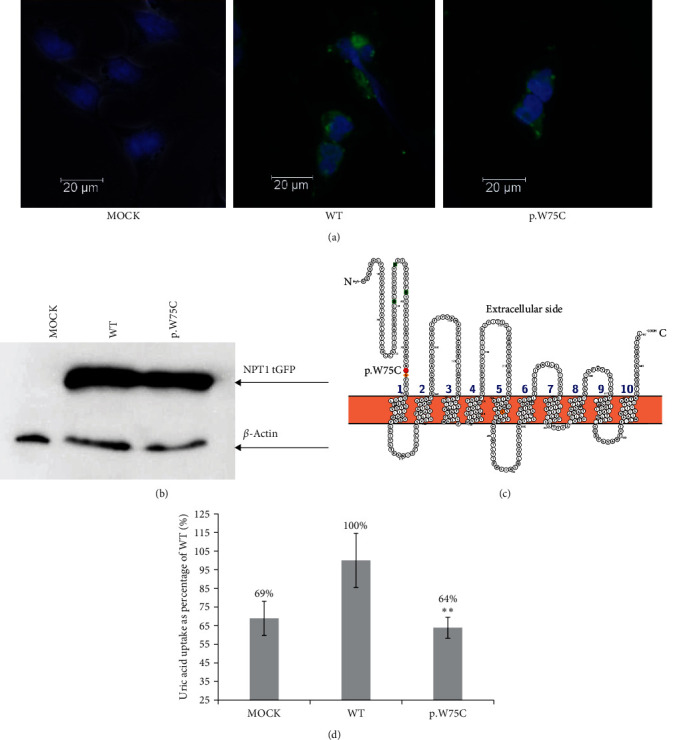
(a) Expression of NPT1 (*SLC17A1*) and its allelic variants in HEK293A cells line. Wild-type protein was tagged with a C-terminal tGFP tag, and cells were transiently transfected by polyethyleneimine lipofection. Samples were fixed with 4% paraformaldehyde, and pictures were taken with a confocal microscope at 400x magnification. Plasma membrane localization was observed in all variants. Cell nuclei were stained with DAPI (blue). (b) Western blot of NPT1 allelic variants. Expression of the protein was detected with tGFP antibody and anti-*β*-actin antibody as a loading control. (c) Predicted structure of NPT1 protein visualized in Protter [58]. Allelic variants are marked with red dots. (d) Uptake study with 30 *µ*M 14C uric acid with 30-min incubation. The intracellular uptake of urate is expressed as a % of the wild-type uptake, *n* = 3,  ^*∗∗*^means *p* ≤ 0.01 (Student's *t*-test).

Finally, we performed an uptake assay of the allelic variants in the NPT1 transporter (Table 2). We incubated the cells with 30 *µ*M 14C labeled uric acid for 30 min at 37°C in depolarizing HBSS buffer (Figure 4(d)). We chose urate uptake in the wild type (100%) as a baseline. Uptake by the negative control (MOCK) was 68.9% of the wild type, and as in the other two cases, we suspect that it is caused by endogenous urate transporters or passive flow across the cell membrane. We identified only one allelic variant, p.W75C in this transporter. We found that uric acid transport by NPT1 with this substitution was significantly decreased to 63.9% of the wild type with a *p*-value <1%. This value is similar to an uptake value of the negative control (MOCK). Subsequently, we performed a fluorescent microscopy analysis of protein localization (Figure 4(a)). We observed mainly a cytoplasmic membrane localization in the wild type and p.W75C variant. Western blot analysis of expression (Figure 4(b)) shows that the wild type and p.W75C are expressed in a similar amount of protein, and in the MOCK, no signal was observed. Finally, we examined the evolutionary conservation of p.W75, and we found that tryptophan at this position is conserved among model species of primates, ruminants, artiodactyla, and birds (Figure S1).

## 4. Discussion

Many experimental or clinical works have been published which identify allelic variants in urate transporters in kidney. Most important (and simultaneously well researched) seem to be polymorphisms in transporters ABCG2 [14, 16, 17], GLUT9 [19–21], and URAT1[25–27].Therefore in our previous study, we examined novelty SNP in *SLC22A6* (OAT1) and *SLC22A8* (OAT3) transporters [28]. Now, we focused on new variant identification in not too much researched transporters OAT4, OAT10, and NPT1. We found allelic variant p.P519L in Patient 1 (Table 3), significantly decreased urate transport capacity (Figure 2(d)). This patient was homozygous at ABCG2 (p.Q141K) and SLC2A9 (p.P350L). The p.Q141K variant in the ABCG2 transporter significantly increases the risk of gout[16, 59–61]. It was shown that this substitution significantly decreases urate efflux from cells[15]. We suspect that decreased urate excretion by ABCG2 p.Q141K in Patient 1 (Table 3) may cause gout despite reuptake transporter OAT4 with p.P519L being decreased. The homozygous mutation p.P350L in GLUT9 (*SLC2A9*) probably has little or no effect on urate uptake or efflux, respectively [31]. We found that the p.V202M substitution in OAT4 had no effect in our *in vitro* uptake assay. This variant was present in Patient 2 (Table 3) with hyperuricemia; they were homozygous for p.G25R in GLUT9a (long isoform, Table 3). This GLUT9 variant did not significantly decrease urate transport, nor did it significantly affect gout risk [31, 62]. These facts may explain why the p.V202M variant caused only hyperuricemia and not gout. A similar situation appeared with a p.R343L variant in OAT4, which did not affect transport in our uptake assay. Patient 3 with this variant was homozygous for p.Q141K in the ABCG2, which decreased urate efflux [15]. Two patients with the p.R16H variant in OAT10 have gout; reuptake by this transporter was significantly decreased. It is possible that the main reuptake transporter URAT1 is upregulated when the other reuptake transporters OAT4 or OAT10 have allelic variants, which decrease transport activity such as p.P519L (OAT4) or p.R16H (OAT10). This hypothesis is supported by the decreased FEUA (Table 2) in patients with mutations that significantly decreased transport (Patients 1, 4, and 5; Table 3). We suggest that the scaffold protein PDZK1 may play a regulatory role. PDZK1 interacts with URAT1 at its C-terminus and affects urate transport [63]. The same scaffold protein interacts with the OAT4 transporter and modulates its activity similarly [64]. Direct evidence of an interaction between PDZK1 and OAT10 is currently unavailable. We found the PDZK binding motif at the C-terminus of NPT1 transporter (peptide position 465-TRL-467), but we have no experimental evidence, that this motif really have role in regulation by PDZK1 protein (Figure S1). However, we suspect this interaction is possible because the sequence homology between OAT10 and OAT4 is 32.4% (35.1% for URAT1; data not shown). Allelic variant p.R102H had a negligible effect on urate transport by OAT10, so we suspect that the hyperuricemia in Patient 6 was caused predominantly by heterozygous mutation p.Q141K in ABCG2. Substitution of p.W75C in NPT1 significantly decreased urate transport in our study (Figure 4(d)). It is not surprising that Patient 7 with this allelic variant had higher SUA and hyperuricemia but did not have gout because ABCG2 probably compensated for the efflux of urate. This is supported by a mild decrease in FEUA levels (Table 2). The variant p.I269T in *SLC17A1* increased urate transport and reduced the risk of gout, which shows the effect of this transporter on SUA [48]. Other possible explanation is influence of intestinal uric acid transporters such as *ABCC4* or *SLC17A4* [65, 66]. Patient 7 is homozygous for three allelic variant in this transporter, which are expressed in intestinal epithelial cells. Similarly, intestinal urate transporter *SLC17A4* can affect SUA. We speculate that Patient 7 should have some mutation in intestinal transporter (*SLC17A4* or other), which caused hyperuricemia without change of FEUA.

We next discuss the impact of our described allelic variant on the molecular function of the transporters. We found that substitution of p.P519L significantly decreased urate uptake into HEK293A cells that transiently expressed OAT4 with this variant. The expression and localization of the protein were not changed. P519 is evolutionarily conserved in apes and pigs. We speculate that this mutation, which caused the insertion of leucine (instead of proline) between p.L518 and p.L520, causes a conformation change. Deleting the whole C-terminus of the OAT1 transporter (OAT1 is 42.1% identical with OAT4, data not shown) completely abolishes para-aminohippurate transport. Substitution of p.L512 in OAT1 leads to loss of its function (this leucine is analogical to L520 in OAT4) [67]. Therefore, it is possible that p.P519 can have a similarly important role in human OAT4.

We did not observe any change in protein localization and processing, so we suspect that P519 could function in attaining the correct conformation. We suspect that the function of the C-terminal region of OAT4 will need to be described in more detail. Substitution of p.V202M did not affect our transport assay. In rats, the fourth transmembrane helix in the OCT1 (organic cation transporter *SLC22A1*, 30.5% identity) transporter was shown to be necessary for substrate transport [68]. However, it seems that a valine by methionine (amino acids with similar chemical properties) substitution did not affect the conformation of the helix or the interaction with urate as a substrate.

Similarly, substituting a positively charged p.R343 with a nonpolar leucine did not affect urate transport. The amino acid in this position is located at the carboxy end of a cytoplasmic loop. It seems that p.R343 is not critical for urate handling. In homologous OAT1, a substitution of p.R293W a cytoplasmic loop did not affect para-aminohippurate transport [69]. In OAT10, we identified that the substitution of p.R16H affected uric acid transport (81.8% of wild-type level). R16 is located in the N-terminus of OAT10 (Figure 3(c)) and is conserved in mammal orthologues (data not shown). In human OAT1, the substitution of p.F16Q or p.Q17A decreases the transport of para-aminohyppurate [70]. p.F16 and p.Q17 are conserved in OAT1 and OAT10 (data not shown). The substitution of p.V13M in human OAT1 does not affect estrone sulfate transport [71]. We suspect R16 in OAT10 affects urate binding at the N-terminus. Substitution of p.R102H had an insignificant effect on urate transport. Substitution of amino acids with similar properties also did not affect urate transport. R102 is located in an extracellular loop. Mutation p.P104L in the same loop in OAT1 did not affect para-aminohyppurate transport [69]. Substitution of p.W75C completely abolished NPT1 transporter function. Tryptophan is conserved among ape, mammal, and bird models (Figure S1). This substitution is located in an extracellular loop of the protein. In the Glut 9 transporter, W110 is crucial for urate transport [72]. We hypothesize that p.W75 in NPT1 may be an analog playing the same role in urate transport. We detected five allelic variants in *SLC22A12* (URAT1) transporter, but all these variants are synonymous in protein sequence, so we supposed that these variants are no protective effect for hyperuricemia or gout (Table 3). Further detailed studies concerning urate transporters and their interactions could clarify genetic and molecular background of renal urate transport. In future work, it will be beneficial to study a more comprehensive *in vitro* model to study the interactions of transporters and their variants with other cellular proteins and regulatory elements specific to epithelial cells of the proximal tubule of the human kidney.

## 5. Conclusions

In summary, our findings will deepen our understanding of urate transport-related gout/hyperuricemia risk and the biochemical characteristics of the OAT4, OAT10, and NPT1 transporters. Our identification and functional characterization of rare variants provide a better understanding of renal urate handling systems and support the “Common Disease, Multiple Common, and Rare variant” hypothesis [73], which argues that genetic susceptibility to common diseases, such as gout, does not reside in common genetic variants but rather in a multiplicity of individual rare genetic variations each with relatively high penetrance.

## Figures and Tables

**Table 1:** Human kidney proximal tubule transporter and its allelic variants, which was previously published in our papers.

Gene	Kidney transporter	SNP ref.	Amino acid substitution	Membrane localization	Urate uptake	References
*ABCG2*	ABCG2	rs2231137	p.V12M	?	+	[17]
		rs2231142	p.Q141K	?	−	[17]
		rs372192400	p.R147W	−	?	[17]
		rs753759474	p.T153M	+	−	[17]
		None	p.I242T	+	−	[29]
		rs750972998	p.K360del	+	+	[17]
		rs752626614	p.F373C	+	−	[17]
		rs199854112	p.T421A	+	+	[17]
		rs769734146	p.T434M	+	−	[17]
		None	p.S476P	+	−	[17]
		rs200894058	p.S572R	−	?	[17]
		rs34783571	p.D620N	+	+	[17]
*SLC22A12*	URAT1	rs144328876	p.R92C	?	−	[18]
		None	p.R203C	?	−	[18]
		rs150255373 ^*∗*^	p.P325W	−	−	[30]
*SLC17A1*	NTP1	rs149708935	p.W75C	+	−	This paper
*SLC22A13*	OAT10	rs121908321	p.R16H	+	−	
		rs113229654	p.R102H	+	+	
*SLC22A11*	OAT4	rs201209258	p.V202M	+	+	
		rs75933978	p.R343L	+	+	
		rs144573306	p.P519L	+	−	
*SLC22A6*	OAT1	rs146282438	p.A190T	+	+	[28]
		rs11568627	p.P104L	+	+	[28]
*SLC22A8*	OAT3	rs45566039	p.R149C	+	−	[28]
		rs11568486	p.V448F	+	+	[28]
		rs145474422	p.R513Q	+	+	[28]
*SLC2A9*	GLUT9	rs6820230	p. A17T	+	+	[31]
		rs2276961	p.G25R	+	+	[31]
		None	p.G72D	?	−	[18]
		None	p.I118HfsX27	?	−	[18]
		rs144196049	p.V169M	+	+	[31]
		None	p.G261R	?	−	[18]
		rs112404957	p.T275M	+	+	[31]
		rs73225891	p.D281H	+	+	[31]
		rs16890979	p.V282I	+	+	[31]
		rs3733591	p.R294H	+	+	[31]
		None	p.N333S	?	−	[18]
		rs2280205	p.P350L	+	+	[31]

Correct membrane localization is marked with the symbol “+”, impaired localization is marked with “-”, unexamined localization is marked with “?”. Transport capacity for urate without significant change is marked with “+”, changed capacity is marked with “−”. Unknown influence on localization or transport is marked with “?”.

**Table 2:** Identified nonsynonymous *SLC22A11*, *SLC22A12*,and *SLC17A1* allelic variants, their mutant allele frequency (MAF) and overview of main biochemical parameters in the patient cohort.

Gene	Reference SNP number	Position CDS	Position AA	Variant allele hetero/homozygotes	Allelic variant MAF	Normo-uricemia control MAF	European MAF	Gender (patient identification)	Diagnosis	Familial occurrence	Age at examination (years)	Gout/hyperuricemia onset (years)	BMI at examination (−)	SUA without medication (*µ*mol/L)	SUA with medication (*µ*mol/L)	FEUA without medication (%)	FEUA with medication (%)
*SLC22A11*	rs144573306	c.1556C > T	p.P519L	1/0	0.004	N/A	0.001	Male (Patient 1)	Gout	No	18	16	27	655	300	4.31	1.64
*SLC22A11*	rs201209258	c.604G > A	p.V202M	1/0	0.004	0.003	0	Male (Patient 2)	Hyperuricemia	N/A	35	N/A	N/A	N/A	N/A	N/A	N/A
*SLC22A11*	rs75933978	c.1028G > T	p.R343L	1/0	0.004	0	0.002	Male (Patient 3)	Hyperuricemia	No	21	17	23	463	N/A	N/A	4.92
*SLC22A13*	*rs72542450*	c.47G > A	p.R16H	2/0	0.007	0.009	0.008	Female (Patient 4)	Gout	No	73	73	32	606	N/A	0.75	N/A
								Male (Patient 5)	Gout	No	78	76	24	387	332	3.24	5.02
*SLC22A13*	rs113229654	c.305G > A	p.R102H	1/0	0.004	0.003	0.008	Male (Patient 6)	Hyperuricemia	No	61	58	32	467	483	2.76	5.14
*SLC17A1*	rs149708935	c.225G > T	p.W75C	1/0	0.004	N/A	0.001	Male (Patient 7)	Hyperuricemia	Yes	24	14	24	433	N/A	5.1	N/A

Only in this, patients were detected new allelic variants. *Abbreviations*: BMI, body mass index; SUA, serum uric acid level (*µ*mol/L); FEUA, fraction excretion of uric acid (%).

**Table 3:** Current occurrence of allelic variants in different transporters in the patient cohort.

Variants in other genes	Gene	*ABCG2*	*SLC2A9*	*SLC2A9*	*SLC2A9*	*SLC2A9*	*SLC17A1*	*SLC17A3*	*SLC17A3*	*SLC22A8*	*ABCC4*	*ABCC4*	*ABCC4*	*ABCC4*	*SLC22A12*	*SLC22A12*	*SLC22A12*	*SLC22A12*	*SLC22A12*
	Reference SNP number	rs2231142	rs2276961	rs16890979	rs3733591	rs2280205	rs1165196	rs1165165	rs56027330	rs45566039	rs11568658	rs2274406	rs1678339	rs1751034	rs3825017	rs3825016	rs11231825	rs1630320	rs7932775
	AA change	p.Q141K	p.G25R	p.V282I	p.R294H	p.P350L	p.T269I	p.A100T	p.G279R	p.R149C	p.G187W	p.R317S	p.L904F	p.K1116N	p.N82 =	p.H86 =	p.H142 =	p.A416 =	p.L437 =
Variants in *SLC22A11*, *SLC22A13*, and *SLC17A1*	p.P519L (Patient 1)	**HM**	**HT**	**HT**	wt	**HM**	wt	wt	wt	wt	wt	**HM**	**HM**	**HM**	**wt**	**HT**	**HT**	**HM**	**HT**
	p.V202M (Patient 2)	wt	**HM**	wt	**HT**	wt	wt	wt	wt	wt	wt	**HM**	**HM**	**HM**	**wt**	**HT**	**HT**	**HM**	**HT**
	p.R343L (Patient 3)	**HM**	**HT**	wt	**HT**	**HT**	**HT**	wt	wt	wt	wt	**HM**	**HM**	**HM**	**wt**	**HT**	**HT**	**HM**	**wt**
	p.R16H (Patient 4)	wt	**HM**	wt	wt	**HM**	**HT**	wt	wt	**HT**	wt	**HT**	**HM**	wt	wt	wt	wt	HM	HM
	p.R16H (Patient 5)	wt	**HM**	wt	**HT**	wt	wt	wt	wt	wt	wt	**HM**	**HM**	wt	wt	HM	HM	HM	wt
	p.R102H (Patient 6)	**HT**	**HM**	wt	**HT**	**HM**	**HT**	**HT**	**HT**	wt	wt	wt	**HM**	**HM**	**wt**	**wt**	**wt**	**HM**	**HM**
	p.W75C (Patient 7)	wt	**HM**	wt	**HT**	**HT**	**HT**	**HT**	wt	wt	**HT**	**HM**	**HM**	**HM**	**wt**	**wt**	**wt**	**HM**	**HT**

Only in this, seven patients were detected this new allelic variants, but some other allelic variants in other urate transport proteins, which was described in the previous works. Wt, wildtype; HM, homozygous; and HT, heterozygous.

## Data Availability

The data used to support the findings of this study are available from the corresponding author upon request.

## Abbreviations

FEUA: Fraction excretion of uric acid
HBSS: Hankʼs balanced salt solution
HEK293-A: Human embryonic kidney cell line
NPT: Sodium-dependent phosphate transport protein
OAT: Organic anion transporter
PEI: Polyethyleneimine
SLC: Solute carrier
SNP: Single nucleotide polymorphism
SUA: Serum uric acid level
RPTEC: Primary renal proximal tubule epithelial cells
MDCK: Madin–Darby canine kidney cells.

## Additional Points

*Limitations*. Although the size of the cohort is sufficient, rare, and extra-rare variants that impact the development of hyperuricemia/gout may still be missed. Our uptake assays were limited by the use of the HEK293A cell line, which was transiently transfected by study genes. In future studies, it would be better to use a cell line derived from renal epithelial cells. We have no model for study the interaction of uric acid transporter and its allelic variation. For examining the interaction we would need an epithelial cell line (such as primary renal proximal tubule epithelial cells, RPTEC or Madin–Darby canine kidney cell, MDCK) which will be transfected by the examined transporters with certain allelic variants and with a combination of different variant. It will be very difficult to prepare this experimental model, which will be very artificial and will be loaded by big experimental error. From similar reasons, it would be difficult and artificial to use some *in vivo* model, such as mouse or rat with inhibited uricase activity. The mutual interaction of transporters exceeds the main goal of our paper (bring the first view on new allelic variants and its clinical phenotype).

## Supplementary Materials

Supplementary Materials Table S1: sequences of primers used for site-directed mutagenesis. Table S2: prediction using different models for the allelic variants in the patient cohort. Figure S1: evolutionary comparison of variants in transporters.
