# Supplementary material for: Examining the Association of Rare Allelic Variants in Urate Transporters SLC22A11, SLC22A13, and SLC17A1 with Hyperuricemia and Gout
Source: Dis Markers. 2024 Jan 6;2024:5930566. doi: 10.1155/2024/5930566 (PMC10787658; doi:10.1155/2024/5930566)
Supplement: Supplementary Materials — Table S1: sequences of primers used for site-directed mutagenesis. Table S2: prediction using different models for the allelic variants in the patient cohort. Figure S1: evolutionary comparison of variants in transporters. [file 5930566.f1.docx]

Supplementary

**Title:** Examining the association of rare allelic variants in urate transporters *SLC22A11*, *SLC22A13*, and *SLC17A1* with hyperuricemia and gout

Jiří Vávra, Kateřina Pavelcová, Jana Mašínová, Lenka Hasíková, Eliška Bubeníková, Aneta Urbanová , Andrea Mančíková, and Blanka Stibůrková

1. **Supplementary table**

| SNP | Forward primer | Reverse primer |
| --- | --- | --- |
| *SLC22A11* p.V202M | GGCCTGCGGTTCATGGCCGCTTTTGGGATG | CATCCCAAAAGCGGCCATGAACCGCAGGCC |
| *SLC22A11* p.R343L | GCGTGCCCGTGCTCCTCTGGAGGAGCTGCGC | GCGCAGCTCCTCCAGAGGAGCACGGGCACGC |
| *SLC22A11* p.P519L | GACCCAGGGACTTCTGCTCCCTGACACTATC | GATAGTGTCAGGGAGCAGAAGTCCCTGGGTC |
| *SLC22A13* p.R16H | GAAATAGGTGACTTTGGTCACTTCCAGATACAGC | GCTGTATCTGGAAGTGACCAAAGTCACCTATTTC |
| *SLC22A13* p.R102H | GACATCCTCAGCCACCACTTCAATGAGACGCAGC | GCTGCGTCTCATTGAAGTGGTGGCTGAGGATGTC |
| *SLC17A1* p.W75C | CCCTATGTATAATGGCAGCCCAGATATCCAGG | CCTGGATATCTGGGCTGCCATTATACATAGGG |

**Tab. S1.** Sequences of primers used for site-directed mutagenesis.

| **Gene** | **Reference SNP Number** | **Position CDS** | **Position  AA** | **SIFT** | **PolyPhen** | **CADD** | **REVEL** | **MetaLR** | **MutationAssessor** |
| --- | --- | --- | --- | --- | --- | --- | --- | --- | --- |
| *SLC22A11* | rs201209258 | c.604G>A | p.V202M | 0.02  (deleterious) | 0.08  (benign) | 0  (likely benign) | 0.222  (likely benign) | 0.162  (tolerated) | 0.123  (neutral) |
| *SLC22A11* | rs75933978 | c.1028G>T | p.R343L | 0.07  (tolerated) | 0.898  (possibly damaging) | 19  (likely benign) | 0.188  (likely benign) | 0.283  (tolerated) | 0.236  (low) |
| *SLC22A11* | rs144573306 | c.1556C>T | p.P519L | 0.03  (deleterious) | 0.6  (possibly damaging) | 22  (likely benign) | 0.309  (likely benign) | 0.516  (damaging) | 0.791  (medium) |
| *SLC22A13* | rs72542450 | c.47G>A | p.R16H | 0.42  (tolerated) | 0.022  (benign) | 16  (likely benign) | 0.144  (likely benign) | 0.133  (tolerated) | 0.875  (medium) |
| *SLC22A13* | rs113229654 | c.305G>A | p.R102H | 0.09  (tolerated) | 0.003  (benign) | 16  (likely benign) | 0.06  (likely benign) | 0.132  (tolerated) | 0.424  (low) |
| *SLC17A1* | rs149708935 | c.225G>T | p.W75C | 0  (deleterious) | 1  (probably damaging) | 31  (likely deleterious) | 0.467  (likely benign) | 0.411  (tolerated) | 0.941  (high) |

**Tab. S2.** Prediction using different models for the allelic variants in the patient cohort.

1. **Supplementary figures**


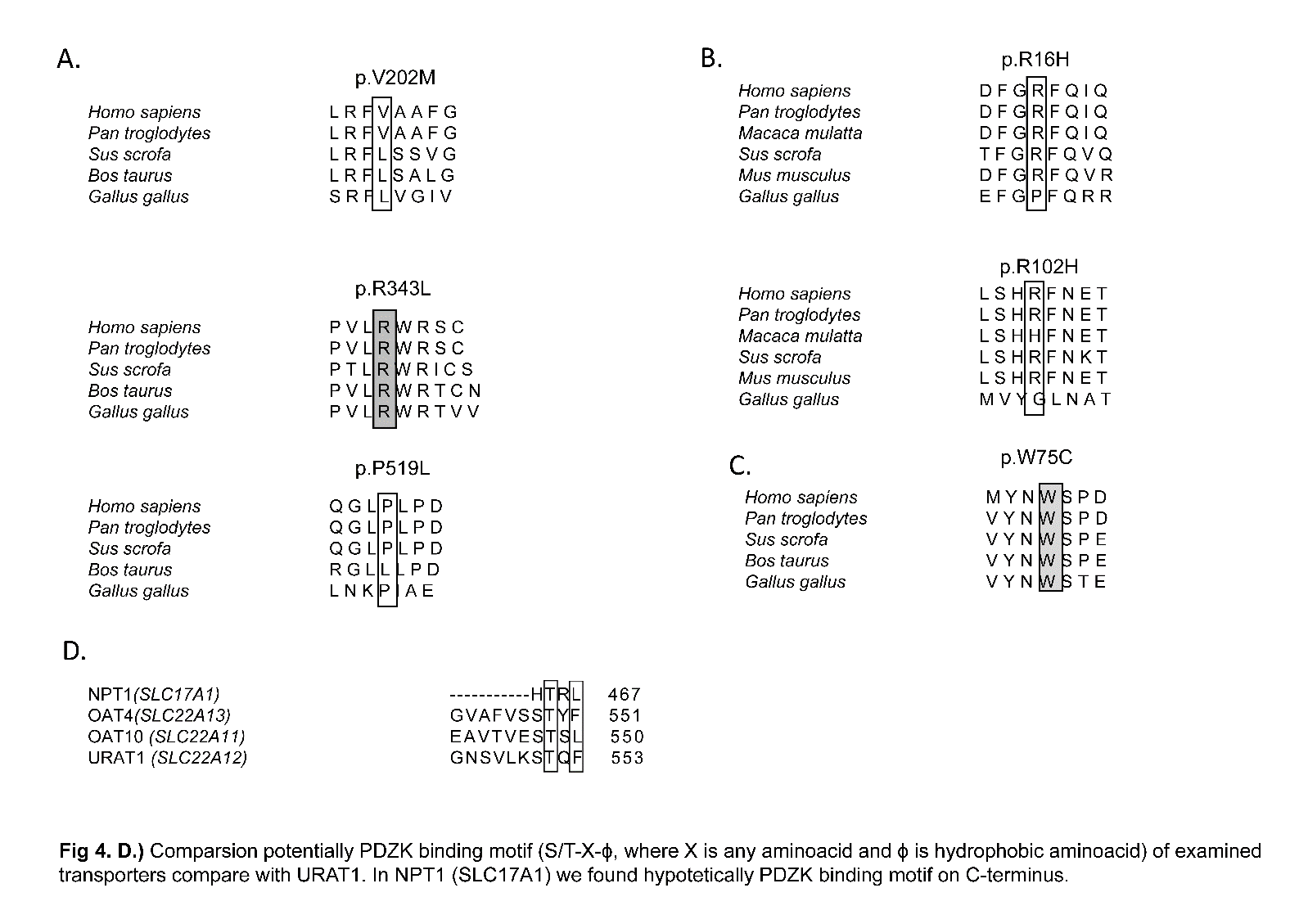


**Fig. S1. A.)** OAT4 (*SLC22A11*) evolutionarily conserved amino acids among seven mammalian species. The position of nonsynonymous substitutions among seven species is marked with grey fill. Identity with human OAT4 *Pan troglodytes* 99.6%, *Sus scrofa 59.3*%, *Bos taurus* 62.6%, *Gallus gallus* 22.9%. **B.)** OAT10 (*SLC22A13*) evolutionarily conserved amino acids among seven mammalian species. The position of nonsynonymous substitutions among seven species is marked with grey fill. Identity with human OAT10 *Pan troglodytes* 99.4%, *Macaca mulatta* 95.4%, *Sus scrofa 77.1*%, *Mus musculus* 73.3%, *Gallus gallus* 43.3%. **C.)** NPT1 (*SLC17A1*) evolutionarily conserved amino acids among seven mammalian species. The position of nonsynonymous substitution among seven species is marked with grey fill. Identity with human NPT1 *Pan troglodytes* 98.5%, *Sus scrofa 63.6*%, *Bos taurus* 61.2%. **D.)** Comparison potentially PDZK binding motif (S/T-X-ϕ, where X is any aminoacid and ϕ is hydrophobic aminoacid) of examined transporters compare with URAT1. In NPT1 (SLC17A1) we found hypothetically PDZK binding motif on C-terminus. Alignment was performed with the Uniprot alignment tool (56).
